# Supplementary material for: The causal role of circulating inflammatory markers in osteoporosis: a bidirectional Mendelian randomized study
Source: Front Immunol. 2024 Jul 18;15:1412298. doi: 10.3389/fimmu.2024.1412298 (PMC11291241; doi:10.3389/fimmu.2024.1412298)
Supplement: Supplementary file 1 [file DataSheet_1.zip › supplementary material/OSTEOPOROSIS-CIM/GCST90274795.tsv.gz_leave_one_out.pdf]

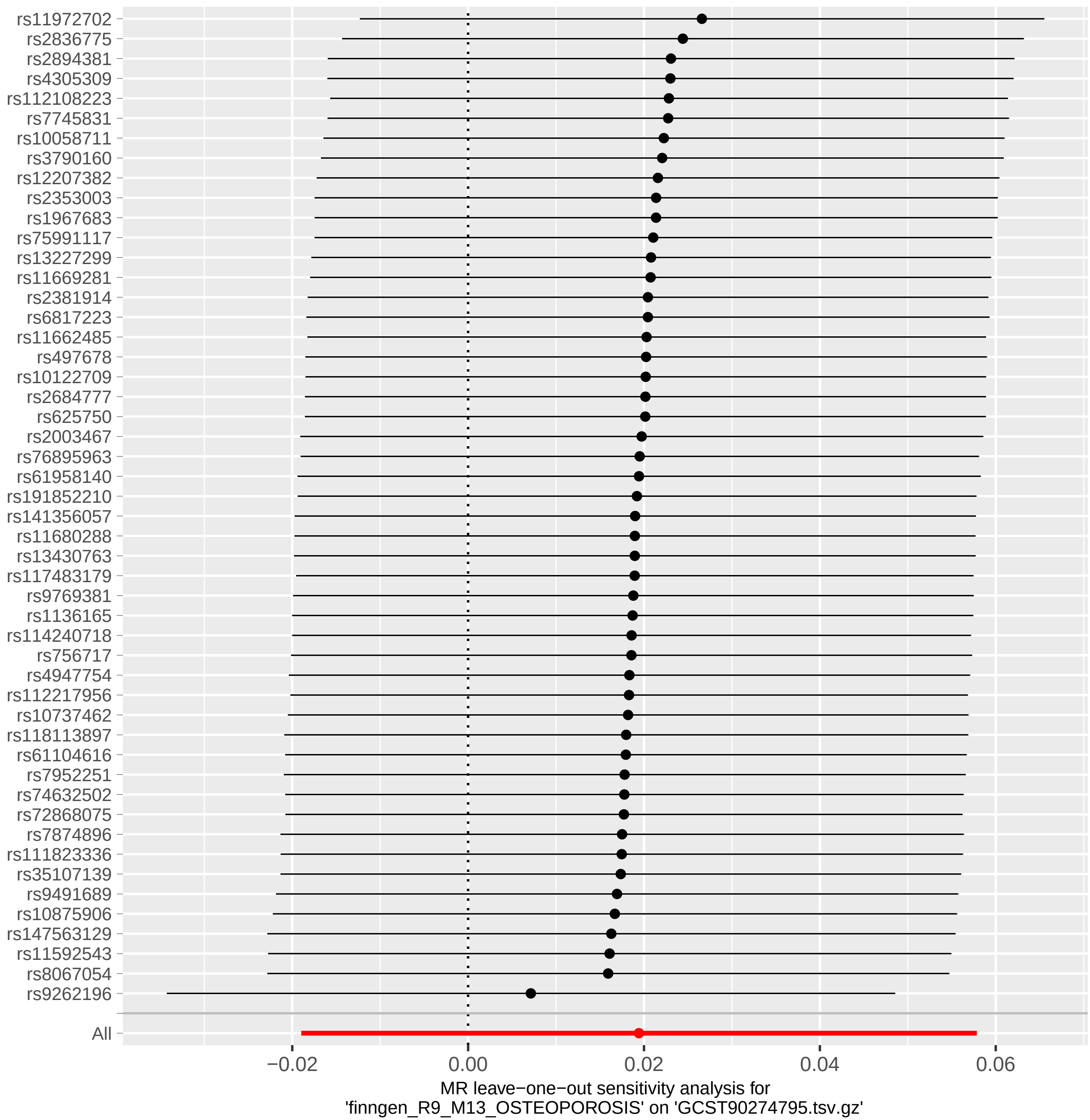
rs11972702
rs2836775
rs2894381
rs4305309
rs112108223
rs7745831
rs10058711
rs3790160
rs12207382
rs2353003
rs1967683
rs75991117
rs13227299
rs11669281
rs2381914
rs6817223
rs11662485
rs497678
rs10122709
rs2684777
rs625750
rs2003467
rs76895963
rs61958140
rs191852210
rs141356057
rs11680288
rs13430763
rs117483179
rs9769381
rs1136165
rs114240718
rs756717
rs4947754
rs112217956
rs10737462
rs118113897
rs61104616
rs7952251
rs74632502
rs72868075
rs7874896
rs111823336
rs35107139
rs9491689
rs10875906
rs147563129
rs11592543
rs8067054
rs9262196
All
−0.02
0.00
0.02
0.04
0.06
MR leave−one−out sensitivity analysis for
'finngen_R9_M13_OSTEOPOROSIS' on 'GCST90274795.tsv.gz'
